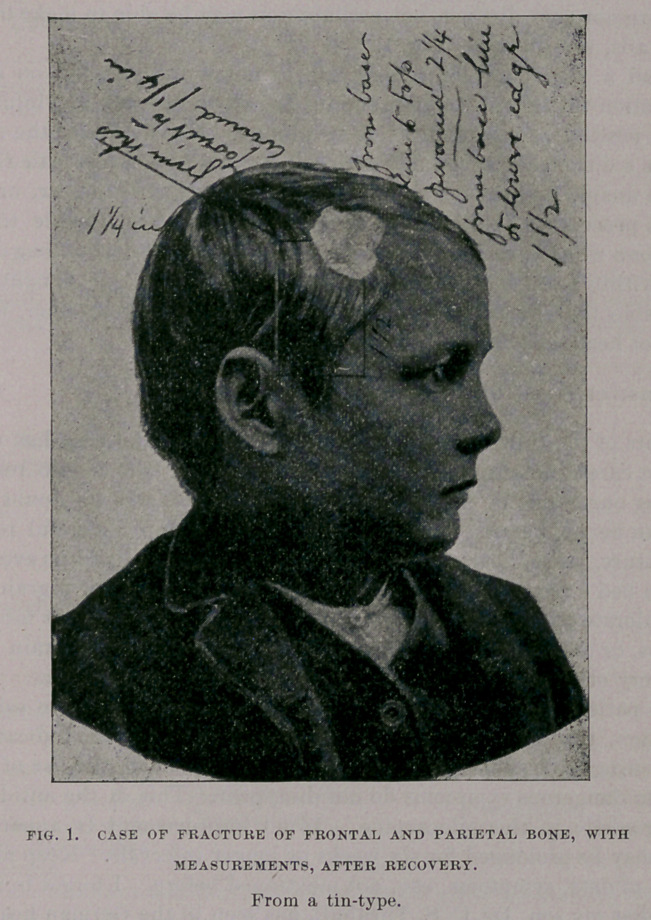# Fracture and Concussion (Contre-Coup) of the Temporal Bone as a Cause of Deafness

**Published:** 1890-08

**Authors:** Laurence Turnbull

**Affiliations:** 1502 Walnut Street; Philadelphia, Pa.


Buffalo Medical and Surgical Journal

VOL. XXX. AUGUST, 1890. No. 1.

### Original Communications.

### FRACTURE AND CONCUSSION (CONTRE-COUP) OF THE TEMPORAL BONE AS A CAUSE OF DEAFNESS.

BY LAURENCE TURNBULL, M. D., Philadelphia, Pa.

WITH REPORT OF CASE AND AN ILLUSTRATION.

THE temporal bone is one of the most important in the ear in its relation to deafness. If it be injured or diseased, it usually involves not only its outer surface, but also the inner wall of the tympanic cavity or middle ear, the mastoid process, and also the internal ear. It is true that we have removed the outer table of the temporal bone in children, the periosteum destroyed by necrosis or caries, or portions of the same in adults, but which, in most cases, was followed by permanent deafness on that side. It is an established fact that the vessels of the diploë of the temporal bone freely communicate with the sinuses in the cranial cavity.

Tucker Randt injected a substance (in the presence of Politzer) into the diploë of the mastoid process, and of the pyramidal portion of the petrous bone, which at once entered into the sinuses of the brain. It has also been found that three-fourths of the abscesses of the brain are in the temporo-sphenoidal lobes.

Fracture of this bone from gun-shot is almost always followed by a profound deafness of one side, but more rarely by *contre-coup* of the other side. A very severe blow on one side will at times be recovered from.

The following is the statement of the case I now present, in a letter from the surgeon, Dr. D. P. Miller, of Huntingdon, Pa.

I take the liberty of asking your opinion of the following case, and whether or not you think it can be benefited:

On January 18, 1889, a boy, aged thirteen years, was struck on the left side of the head by an engine, and the skull fractured near the top. The fracture involved the frontal and parietal bones, but not the temporal. I was called to the case and found the bones detached from the membrane and muscles of the scalp.

I removed the bone, which left an opening in the skull larger than a silver dollar. The wound healed kindly, but from the date of *injury the boy* has been *perfectly deaf.* I do not think there is any injury to the membrane of the ear, and I am disposed to attribute the deafness to the injury of the nerve, but as this is the first case of the kind I have ever met with, and am not familiar with such cases, I may be mistaken. I have had four (4) cases of fracture of the skull, where I removed the bone, with three perfect recoveries, but this is the first case of resulting deafness.

As the case was rare we examined all the authorities on this subject, and found the following reported :

The only mention of fracture of the parietal bone alone in connection with deafness is by Mossakoroski, who found among 1,415 wounded French prisoners but fourteen wounds of the bony parts of the head. Of this number the forehead was the seat of the injury in one case, *the parietal bones* in ten, and the temporal in three cases. In four of the cases severe cerebral symptoms developed, and in four of them hemorrhage from the ear occurred. Objective examination showed that of these fourteen invalids, four were deaf in one ear, whilst two were deaf in both ears, with severe psychical disturbance. Special aural examinations of these patients were apparently not made. [Mossakoroski, Paul. *Deutsche Zeitschrift für Chirurgie, Bund* 1, 1872.]

Desiring a more detailed account of the accident, *contre-coup* of the temporal bone, the following was received from the father and engineer :

On the 18th day of January, David Louder, the young boy who, accompanied by his father, paid you a visit on the 22d of the present month, was struck by the hand-railing of a tender on the back end of a locomotive. He was picked up and carried about 100 feet to his residence in an unconscious state, and remained so until the following Tuesday. After regaining consciousness he could hear for two days, after which and up to the present time he has remained entirely deaf.

The engineer made the following statement:

In regard to the accident to the boy that was brought to you on the 22d inst., I would state that I am of the opinion that there is a bar, with which our engine is equipped for the purpose of a hand hold for the men on the rear end of the tank, having a ball on the end one and one-half or two inches in diameter; this I think struck the boy on the head. I could not say for certain, as I knew nothing of the accident at the time. I would also state that I think I was running at the time about twelve or fourteen miles per hour.

In answer to the Doctor's letter, I stated :

Could you get a photograph of your patient (injury)? And also enter into a little more detail as to measurements, as some of the most vital points are not quite clear, but sufficient has been stated to ground a *most unfavorable prognosis.* There has no doubt been a fracture of one temporal (petrous portion) bone and concussion (contre-coup) hemorrhage on the other

FIG. 1. CASE OF FRACTURE OF FRONTAL AND PARIETAL BONE, WITH MEASUREMENTS, AFTER RECOVERY.

From a tin-type.

side. If the boy was here, an examination would be in order, but as he is not it would not be worth while. Examine for a cicatrix in the membrana tympani; also see if the umbo or bright spot be sunken or lost. Test his hearing with voice, tuning fork, and loud whistle, and two books struck together with force, and write me again.

The following reply was received:

Yours received this A. M. This afternoon the boy called at my office and I had the enclosed tin-type taken. See Fig. The paper pasted on the head is located on that portion of the head from where I removed the bone, but in taking the picture the head is reversed, the wound being on the *left side*, as in picture.

You will notice a line drawn from the ear (when attached) to upper part of Fig. From this line to the back of the wound in the skull it is one and one-quarter inches. From the base line to top of wound is two and one-quarter inches. I tried to make figures giving distances on the plate, but they were not very distinct, but perhaps you may be able to make them out. For details, see picture. Fig. 1.

I had a very poor light to-day to examine the ear, but on a former examination thought the membrane in the right ear was not injured, but was not positive as regards the left membrane. He could hear the clapping of books a distance of seven feet with left ear and fifteen feet with right ear, but was unable to hear watch tick when pressed against the ear, or on the mastoid process, or held between the teeth, and was also unable to hear a shrill bone whistle, such as hunters use in calling their dogs at long distance. When within a few inches of the ear could not hear it. I thought it very remarkable that he could hear the books when struck together, when he could not hear the whistle.

I answered as follows:

Yours of the third received; also the tin-type. The case has received my most careful attention, as well as my son's. I can see no reason for changing our opinion. The concussion of the book was no doubt felt, as there can be no hearing power left, we fear. Keep a careful record of temperature, pulse, heart, and condition of secretions. Give him every night while in bed a hypodermic injection of first one-twentieth of a grain of the hydrochlorate of pilocarpine, for five days, then double it; two tablets for five days, or one-tenth of a grain; and then one-eighth of a grain for five days every other night; then you can further increase the dose.

The patient must be carefully watched; weakness, excessive salivation, palpitation, throbbing, giddiness, and impaired vision are indications for the lessening of the dose, or suspending the treatment, or the use of atropia if certain dangerous symptoms do not disappear. This is the antidote and is antagonistic to the pilocarpine. If no improvement is apparent, the course may be prolonged for six weeks or longer intervals. Keep also particulars of date, symptoms, etc., not mentioned before. I forgot to mention to you that my son, Dr. C. S. Turnbull, has seen in the German hospital, of this city, two or three cases in which the head (temporal region) was squeezed between two solid bodies, and in all the cases deafness was profound and was not benefited by treatment. Borrow a tuning fork from some musician and test the hearing in the air; then over the temporal bone, mastoid, nape of the neck, occipital, and bridge of nose. This is the safest test. It may be felt, but unless there be auditory nerve-power no musical tone can be heard.

The boy was sent to me and the following examination and diagnosis made of the case :

DIAGNOSTIC TABLE.

| | | | |
|---|---|---|---|
| April 22, 1889. | Name, David Louder.<br>Age, 14 years. | Occupation, in shoe factory.<br>Residence, 1419 Penn street, Huntingdon, Pennsylvania. | |
| Duration. | Origin and probable cause. | No pain. | Subjective sounds. |
| History Left. { January 18th.<br>Three months. | Suddenly after 4 days.<br>Trauma. | | Singing. |
| Left. | Bone conduction.<br>Diminished.<br>Disappeared.<br>Negative. | Air conduction.<br>Diminished.<br>Disappeared.<br>Negative. | |
| Right.<br>Meatus<br>Normal. | Membrana<br>Tympanii. | Hearing distance before, and after air douche *nil*. | Bone conduction on Mastoid process and with Tuning Fork. |
| Adherent,<br>Not movable,<br>No Light cone.<br>Hammer handle not seen.<br>Right M. tympani curvature irregular. | Use of Politzer air douche.<br>No improvement in hearing. | Absent. | Absent. |
| Light cone.<br>Color normal.<br>Hammer handle seen.<br>Left M. tympani curvature sunken. | | Absent.<br><br>Only in teeth. | |
| Nostrils: | Left, Normal. | Right, Normal. | |
| MECHANICAL AIDS:<br>The most powerful hearing trumpet, with no sound, also Seigle's apparatus and | Politzer's method,<br>Catheterization. | THERAPEUTICS:<br>Pilocarpine,<br>Ether vapor. | |

The little patient came April 22d, and I devoted all my morning to him, (see table) as well as sending him to Dr. Charles S. Turnbull, and also, for fear anything was omitted, I sent him to my chief assistant at my Clinic at Jefferson College Hospital. I procured the most powerful ear trumpet made, and had him tested with it. I opened the Eustachian tube and then introduced the catheter and injected the vapor of ether into the middle ear, testing the left ear which has had a central perforation, and found both free.

There is evidence of adhesion and deposit of blood in posterior surface of the right drum. Both my son and myself think that there must have been double fracture, or fracture on one side and *contre-coup* (concussion) on the other.

I instructed as follows:

Now, I want you to do three things: fit and insert a gum-tube in the right meatus, and then fit a syringe to it and exhaust carefully each day for a short time (one week) as you see in my book, termed "Seigle's apparatus." Also place him on the pilocarpine, as I have before directed. If no good results follow, touch his system by mercury and iodide of potassium. After all this is done I will be satisfied. He must be sent after a few months, in the Winter, to a deaf-mute school if he continues as deaf as he is, for unless this is done he will lose his power of expressing himself by spoken language, and at home he must only use lip-teaching; no signs, if possible. Tell his father to cut off his moustache, so that he can see the action of his lips and mouth.

The noise of the book was no doubt *felt*, as there can be no hearing power left.

Abstract of doctor's letter:

After our patient returned from the city he had an abscess form in the right ear which discharged very freely, and for a time was quite painful. This prevented my treating the ear as you suggested, but have gotten through with the "Seigle method;" used pilocarpine from one-twentieth up to one-eighth grain.

The bowels have been moved three and four times a day, and the urine largely increased in quantity. The action on the skin is not at all free, as it appears to exert its influence principally on the kidneys. The morning temperature is lower than normal, ninety-seven and two-fifths, and the evening temperature slightly increased, ninety-nine and two-fifths and ninety-nine and four-fifths degrees.

He will increase the injection to one-tenth grain, and subsequently to one-eighth of a grain from May 16th to June 3d.

There was no improvement in the hearing by this careful treatment.

1502 Walnut Street.

[Note.—This paper was prepared for the Nashville meeting (1890) of the American Medical Association, but too late for presentation there.—Editor.]

## Figures and Tables

**Fig. 1. f1:**